# Experimental Study of the Mechanical Properties and Microstructures of Lightweight Toughness Cement-Based Composites

**DOI:** 10.3390/ma12233891

**Published:** 2019-11-25

**Authors:** Wenhua Chen, Zhiyi Huang

**Affiliations:** College of Civil Engineering and Architecture, Zhejiang University, Hangzhou 310000, China; cwh2018@zju.edu.cn

**Keywords:** lightweight, toughness, cenospheres, pozzolanic activity, Microstructures, Nano-indentation

## Abstract

The effects of cenospheres, an industrial waste residue, on the compressive strength, flexural strength, toughness, ductility, chemical component, microstructures, and micromechanics of lightweight toughness cement-based composites (LTCCs) by comprehensive experimental tests are explored in this paper. The results indicate that an increase in the amount of cenospheres leads to a decrease in the compressive and flexural strength of LTCCs. However, the specific strength of LTCCs increases with increasing cenosphere content. LTCCs containing 20% cenospheres and 1% fiber volume have the best toughness and ductility. Significant strain hardening occurs during the four-point bending and uniaxial tensile process. Furthermore, the incorporation of cenospheres promotes the hydration reaction of LTCCs due to its high pozzolanic activity. The LTCC cement paste has a low bonding strength to the fiber, which helps the fiber to be pulled out to produce greater bending deformation and tensile strain. The elastic modulus and hardness of the LTCC cement paste decrease linearly with increasing cenosphere content, which also causes the LTCC microstructure to become loose and more ettringite to generate. The weak interfacial transition zone between the cenospheres and the cement matrix is the important reason for the decreasing compressive strength of the LTCC. In conclusion, LTCC incorporating cenospheres is suitable for long-span steel deck pavements due to its light weight and excellent toughness. The successful application of cenospheres in engineering construction can save natural resources and contribute to sustainable development.

## 1. Introduction

Lightweight concrete has the advantages of light weight, high specific strength, thermal insulation, sound insulation, saves energy, and protects the environment [1,2,3,4,5], so lightweight concrete has been widely used in bridges, buildings, oceans and other engineering applications [6,7]. Lightweight concrete is becoming a hotspot for researchers.

The density of lightweight concrete is generally considered to be in the range of 1400–2000 kg/m^3^ [8], which is lighter than normal concrete (2400 kg/m^3^). Currently, there are two main types of lightweight concrete: (1) lightweight aggregate concrete and (2) foam concrete. Researchers have used various different natural lightweight aggregates to blend into concrete to obtain lightweight concrete with superior performance. Light aggregates generally contain ceramsite, expanded perlite, expanded shale, crumb rubber, expanded polystyrene beads, glass beads, etc. [9,10,11,12]. The lightweight concrete made from the above materials often has some common disadvantages. For example, the mechanical properties and toughness of concrete deteriorate, and the duration of impermeability deteriorates due to an increase in the number of connecting holes.

At present, the cementitious material of traditional concrete is still mainly cement. However, the production of cement emits a large amount of CO_2_, which accounts for about 5–7% of the total global emissions [13]. Therefore, it is important to introduce recycled materials in the cementitious material to replace part of the cement [14,15,16,17], which can reduce CO_2_ emissions and is of great significance to the global environment. Dating back to 1984, Montgomery [18] firstly tried cement paste with a high cenosphere content. Cenospheres are hollow spherical particles that are sifted from the residue of coal-fired power plants. Cenospheres have many superior properties [19] such as low density, high temperature resistance, good dispersibility, good workability, and high strength. Cenospheres are suitable for many building materials applications, and are one of the most valuable by-products of fly ash [20]. In China, more than 600 million tons of fly ash are produced every year, most of which are wasted and landfilled. Therefore, making full use of cenospheres is beneficial in protecting the environment and promoting sustainable development.

In the past decade, many researchers have conducted work on ultra-lightweight cement-based composites that incorporate cenospheres and achieved good research results. Blanco et al. [21] successfully developed a lightweight concrete with cenospheres with a 28-day compressive strength of 27–33 MPa, and found that its strength was much higher than that of lightweight concrete (1–5.5 MPa) that incorporated expanded perlite. Huang et al. [22] successfully added iron ore tailings, fly ash, and fly ash cenospheres to green, lightweight, engineered cementitious composites (GLECC) with a density of 1649–1820 kg/m^3^ and a 28-day compressive strength of 25.0–47.6 MPa, which is beneficial in making full use of waste resources and protecting the environment. Wang et al. [23] investigated ultra-lightweight cement composite incorporated with a shrinkage reducing admixture and steel fiber, which has a low density of 1474 kg/m^3^, high compressive strengths of 68.2 MPa, and high flexural strength of 8 MPa. More importantly, deflection hardening behavior occurred in the bending test.

The above research scholars focused on the mechanical properties of lightweight concrete, but others scholars have done a lot of research work on other properties of lightweight concrete. Liu et al. [24] found that a lightweight concrete (1310 kg/m^3^) with a low silica fume and water-to-cementitious materials ratio could have stronger resistance to chloride-ion penetration and water compared with normal concrete. Du et al. [25] indicated that incorporating nano-silica (within 3%) could improve the permeability, water sorptivity, and chloride diffusion of ultra-lightweight cement composite. Huang et al. [26] developed an ultra-lightweight cement composite reinforced with a small amount of 0.2% PP fiber, which can achieve fire resistance. Compared with normal concrete and normal lightweight aggregate concrete, ultra-lightweight cement composite showed superior compressive properties after exposure to high temperatures (900 °C). Wang et al. [27] developed a novel ultra-lightweight cement composite (ULCC) reinforced with steel fiber with a density ranging from 1250 to 1550 kg/m^3^ and proposed an analytical model to predict the ultimate resistance of the ultra-lightweight cement composite.

Some scholars have done research on special engineering applications of lightweight concrete. Jin et al. [28] suggested that a protective coating on concrete wind turbine towers could use lightweight engineered cementitious composite to enhance durability, which can reduce maintenance costs and extend service life. Yan et al. [29] recommended that ultra-lightweight cement composite be applied to reinforced flat slabs, double-skin-composite (DSC) slabs, and offshore constructions. These studies have shown that lightweight concrete incorporated with cenospheres has good engineering application prospects.

It can be seen that most studies focus on the density and strength of lightweight concrete and the performance of lightweight concrete in terms of durability. However, there is little research on the increased toughness of lightweight concrete and its micromechanical properties.

In this study, the mechanical properties and microstructures of lightweight tough cement-based composites with different cenospheres were studied in order to provide basic data for practical application in the future. First, this paper investigates the mechanical properties of lightweight toughness cement-based composites (LTCCs) that contain cenospheres, including compressive strength, flexural strength, tensile performance, and toughness performance using comprehensive experimental tests. Second, the phase composition of LTCCs was investigated using x-ray diffraction (XRD) tests. Finally, the microstructure and micromechanics of LTCCs were tested and characterized by scanning electron microscopy (SEM) and nano-indentation (NI). The effect of cenospheres on the performance of LTCCs was explained based on the microscopic mechanism.

## 2. Experimental Program

### 2.1. Materials

**Figure 1:**
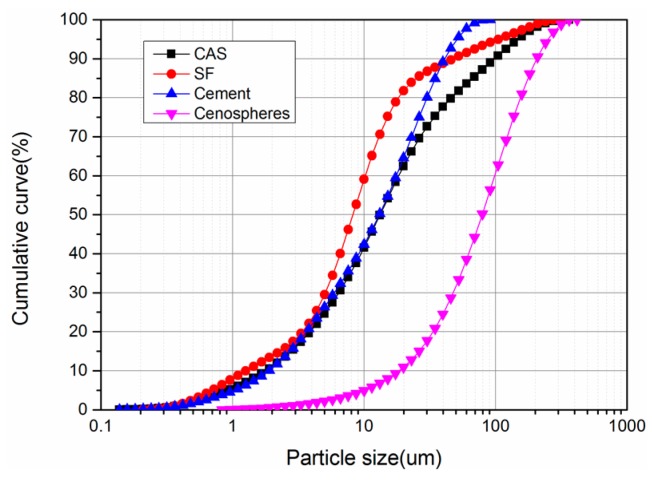
Particle size distribution of powders.

**Figure 2:**
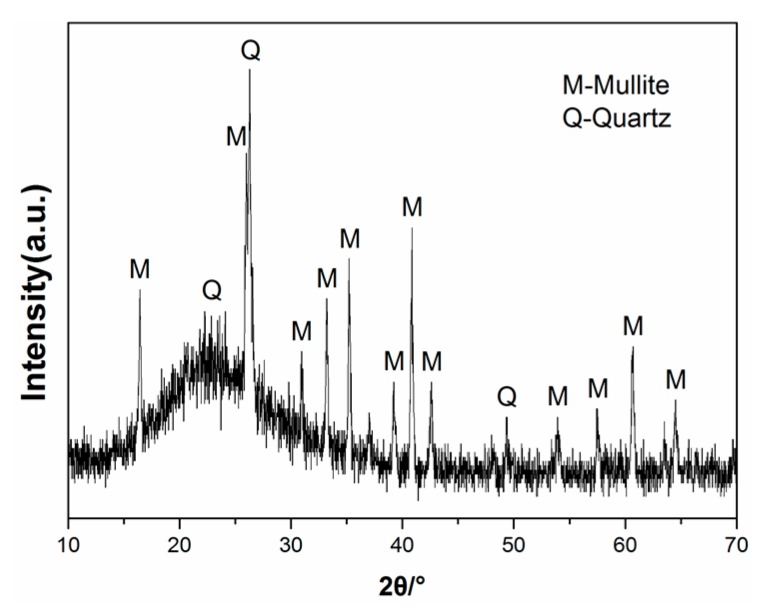
XRD diffractogram of cenospheres.

**Figure 3:**
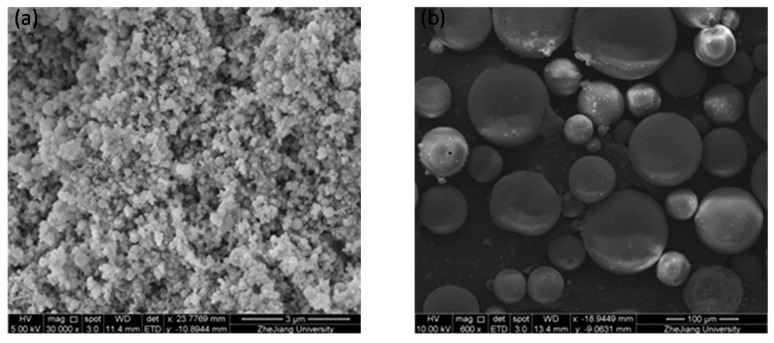
SEM photograph of (**a**) silica fume and (**b**) cenospheres.

The lightweight toughness cement-based composites studied in this paper include cementitious binders (mainly contains Portland cement and silica fume), cenospheres, an expansive agent, a shrinkage-reducing agent (SRA), and polycarboxylate superplasticizer (PSP). Particle size distribution of powders used in the LTCCs is given in Figure 1. Type-II 52.5-R Portland cement, produced by Conch Cement Co., Ltd., Anhui, China, has a specific surface area of 365.3 m^2^/kg, a corresponding compressive strength of 3d, and 28d is 32.1 MPa and 59.4 MPa, respectively. The cenospheres studied in the LTCC have a bulk density of approximately 800 kg/m^3^, with a diameter of 45–300 um, and the average diameter was 100.7um. Figure 2 shows the crystal structure (peaks) obtained using X-ray Diffraction (XRD), and it was found that cenospheres contain two minerals, mullite and quartz. The chemical compositions of PC, cenosphere and silica fume, are shown in Table 1. The diameter and density of the silica fume prepared for the LTCC were about 10 μm and 0.62 g/cm^3^, respectively, and 92.26% of SiO_2_. The micrograph imagery of the silica fume and cenospheres obtained from scanning electron microscopy (SEM) are shown in Figure 3.

To reduce the potential for high shrinkage deformation during hydration [30,31], it is important to incorporate an expansion agent and shrinkage-reducing agent in the LTCC. The calcium sulfoaluminate (CSA) that was used as the expansion agent in the LTCC is produced by Tianjin BaoMing Co., Ltd., Tianjin, China. The shrinkage-reducing agent (SRA) helps the LTCC resist shrinkage cracking. PSP uses a polycarboxylate powder, which is good for improving the rheological properties of the LTCC. To control the cost and the construction workability of the LTCC, the fiber is PVA fiber and its volume is controlled at 1%. Polyvinyl alcohol fiber (PVA fiber, Kuralon K-II REC 15) with a length of 12 mm and a diameter of 39 mm is produced by Kuraray Co., Ltd, Japan. Its tensile strength is 1600 MPa, and weight is 1.3g/cm^3^.

### 2.2. Mix Design and Specimen Preparations

In the mix design of the lightweight toughness cement-based composites, the cenospheres play an important role compared to other components. So this paper mainly investigates the effect that cenospheres have on the mechanical properties and microstructures of the LTCC. Mixture proportions of LTCC are listed in Table 2. The proportion of the matrix was the same in all the samples. The ratios of the cenospheres incorporated in the LTCCs were 10%, 20%, and 30% of the cementitious binders, respectively. Conventional cement-based composites without cenospheres were used as a reference group.

First, the cement, silica fume, cenospheres, expansive agent, and shrinkage-reducing agent were stirred for 3 min until evenly mixed. Second, water and polycarboxylate powder were added during the mixing process. Then the materials were stirred to reach the flow state, fibers were added, and the LTCCs were quickly stirred for 1 min. The LTCCs were poured into the specified molds with vibration. Specimens were demolded after curing 24 h at room temperature and cured at a temperature of 20 °C and a relative humidity of 95%.

### 2.3. Tests Methods

The mechanical tests of the LTCCs included compressive tests, flexural tests, bending toughness tests, and uniaxial tensile tests. The microstructural tests of the LTCCs included X-ray diffraction (XRD) tests, scanning electron microscopy (SEM), and nano-indentation (NI) tests.

#### 2.3.1. Compressive and Flexural Strength of LTCCs

Compressive strength and flexural strength are the most common strengths of cement-based composites. They are often used as the primary performance criteria for characterizing cement-based composite quality. Compressive and flexural tests were conducted to quantify the possible increase or loss of strength of the LTCCs caused by cenospheres. Compressive and flexural strength tests were conducted according to the Chinese specification [32]. The dimension of compression cube specimens and flexural prismatic specimens were 70.7 mm × 70.7 mm × 70.7 mm and 40 × 40 × 160 mm, respectively. At least three repetitive tests of samples were conducted to obtain reliable data, which represented the compressive and flexural strength of LTCC.

#### 2.3.2. Bending Toughness of LTCCs

**Figure 4:**
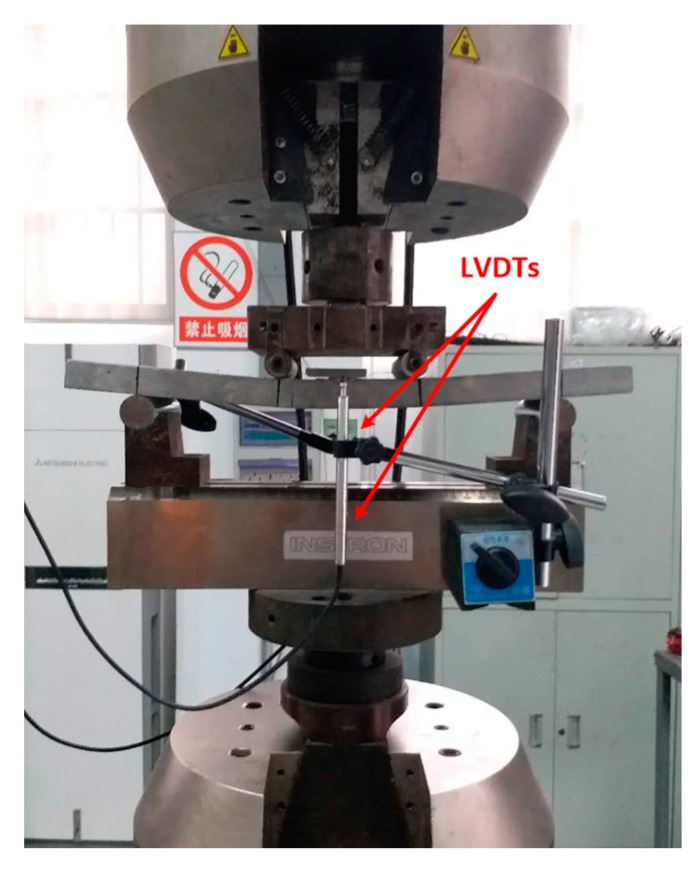
Bending toughness test.

The four-point bending specimens were loaded downward at a speed of 0.2 mm/min by a 250 kN Instron testing machine. The dimension of the thin plate bending specimens was 400 × 100 × 20 mm. The loading span and support span of the test specimens were 100 mm and 300 mm, respectively. The tests used a force sensor and linear variable differential transformers (LVDTs) to collect displacement and force in the middle of the specimens. Two LVDTs were fixed on both sides of the bending specimens, as shown in Figure 4. Experiments determined the force and displacement curves, which were converted into stress-displacement curves using Equation (1).

(1) $$\sigma =\frac{FL}{bh^{2}}$$

where *σ* is the flexural strength (MPa), *F* is the load (N), *L* = 300 mm (the span of the bending specimens), *b* = 100 mm (the width of the bending specimens), and *h* = 20 mm (the height of the bending specimens).

#### 2.3.3. Uniaxial Tensile Properties of LTCCs

**Figure 5:**
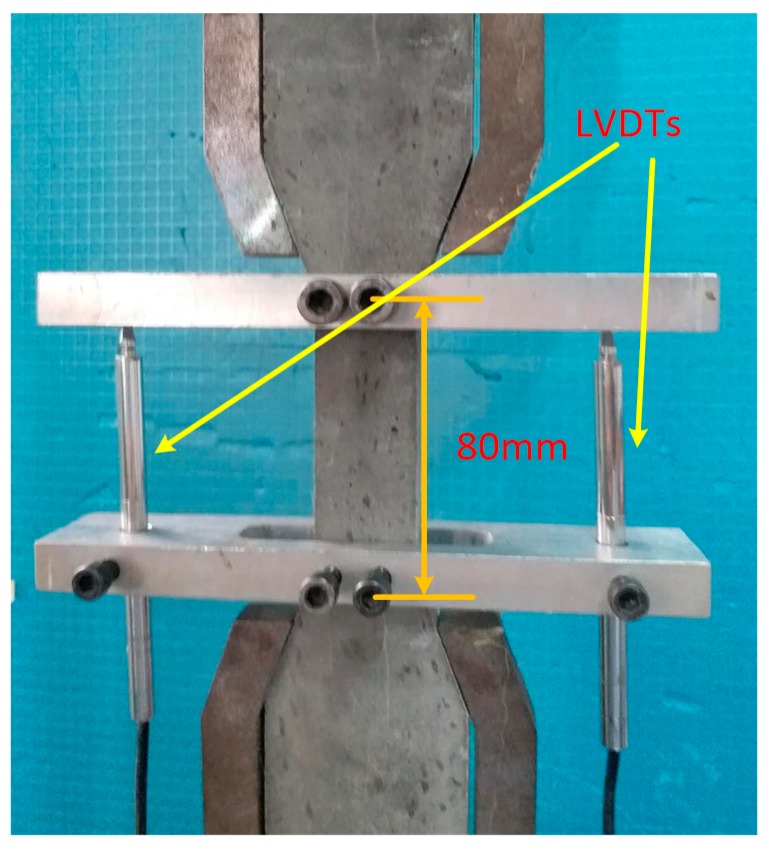
Uniaxial tensile test.

The uniaxial tensile tests of the dog-bone specimens were conducted using a 25-kN Instron testing machine. The distance between the two linear variable differential transformers (LVDTs) was 80 mm. The effective cross-sectional dimension of the dog-bone specimens was 13 × 30 mm. To record the deformation, two LVDTs were mounted on either side of the uniaxial tensile specimen, as shown in Figure 5. The LTCC uniaxial tensile test was conducted at a displacement speed of 0.2 mm/min according to the Japan Society of Civil Engineers (JSCE) [33].

#### 2.3.4. Chemical Composition of LTCCs

To explore the effect of cenospheres on the chemical composition of the LTCC, XRD test was conducted. Small specimens of LTCC were first soaked in absolute ethanol for 24 h and in an oven at 60 °C for 48 h. The test specimens were broken using a hammer and ground into powder. The powders were passed through a 200-mesh sieve.

In this study, XRD (XRD-7000, Shimadzu Corporation, Japan) was utilized to analyze the effect of cenosphere content on the phase structure of LTCCs. The test conditions were accelerated voltage and applied current of 40 kV and 30 mA, respectively. The LTCC XRD pattern was recorded in a range from 10° to 70° in the continuous scanning mode at a rate of 4°/min.

#### 2.3.5. LTCC Microstructure

Field emission scanning electron microscopy was used to observe the microstructural characteristics, hydration products of LTCC samples, and the adhesion of the fiber to the cement matrix in the natural state. The crushed specimens were sputtered with gold under a vacuum. The microscopic morphology of the LTCCs was observed by using FESEM at a voltage of 10 kV.

#### 2.3.6. Nano-Indentation of LTCC

**Figure 6:**
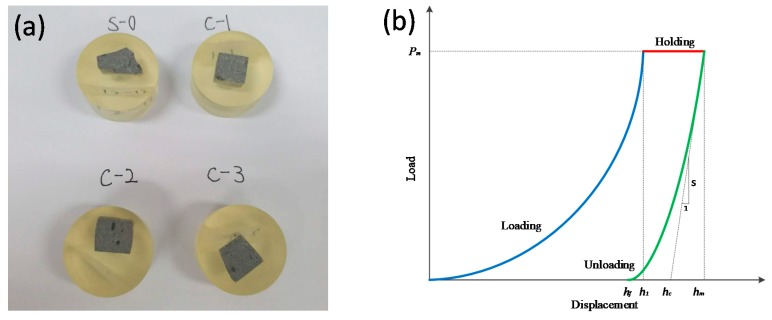
(**a**) Nano-indentation samples. (**b**) Typical nano-indentation loading and unloading curve.

A Nano-indenter was used to investigate the micromechanical properties of hydration products and the interfacial transition zone, which mainly included elastic modulus and hardness. Small particles of the specimens having a diameter of about 1 cm were taken and cold-set with epoxy resin. The samples were sanded and polished by a sander to make the surface smooth. The prepared specimens are shown in Figure 6a. The indenter was a triangular pyramid Berkovich diamond indenter with an angle between the centerline and the cone of 65.03°. During the Nano-indentation test loading process, the holding time was 10 s, the maximum indentation load was set to 100 mN, and the loading and unloading rate was 100 mN/min. To avoid the influence of the distance between the measuring points being too close, the center distance of each measuring point was not less than 3 μm. Force and displacement curves were obtained for each group of experiments.

The Nano-indentation test was conducted by pressing a known hardness and elastic modulus into the unknown material to obtain the load-displacement curve. The mechanical parameters were obtained by analyzing the curve. Figure 6b shows a typical nano-indentation loading and unloading curve, which included notes on important mechanical parameters. In the schematic, hm is the maximum indentation depth, hc is the indentation contact depth, and S is the slope at the top of the unloading curve, which is the contact stiffness. The elastic modulus and hardness of the LTCCs were calculated using the Oliver elastic calculation method [34].

(2) $$E_{r}=\frac{\sqrt{\pi }}{2\beta \sqrt{A}}S$$

(3) $$\frac{1}{E_{r}}=\frac{1-{v_{i}}^{2}}{E_{i}}+\frac{1-v^{2}}{E}$$

(4) $$H=\frac{P_{\max}}{A_{c}}$$

(5) $$A_{c}\approx \alpha {h_{c}}^{2}$$

where *Er* is the modulus of reduction of the material in Equation (2), and A is the contact area between the indenter and the material to be tested. For the Berkovich indenter, the indenter shape factor β was 1.034. For the material true modulus *E* and the reduced modulus *Er*, there is a relationship shown in Equation (3), where *E* and *ν* are the elastic modulus and Poisson’s ratio of the material to be tested. E and *ν_i_* are the elastic modulus and Poisson’s ratio of the indenter, which were 1.141 GPA and 0.07, respectively. In addition, the indentation hardness was calculated by Equation (4). *P* was the maximum load (100 mN). The contact area between the indenter and the material (Ac) was calculated by Equation (5), where α was the parameter related to the shape of the indenter. It was 24.5 for the Berk indenter.

## 3. Results and Discussion

### 3.1. Mechanical Properties

#### 3.1.1. Compressive and Flexural Strength

**Figure 7:**
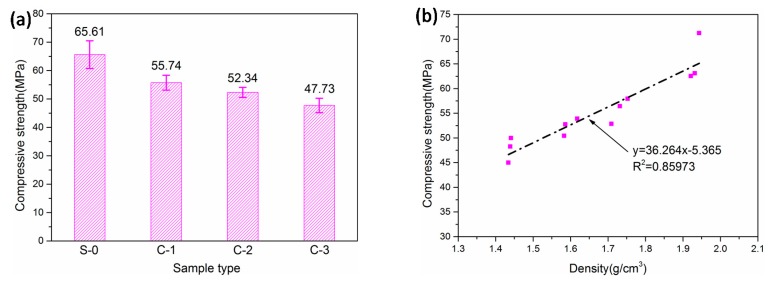
(**a**) Compressive strengths of LTCCs. (**b**) Compressive strength with respect to density.

Figure 7 shows the measured 28-day compressive strengths of the LTCCs and the plots of the compressive strengths with respect to density. S-0 without cenospheres was the reference group. It can be seen that the compressive strengths of the LTCCs decrease from 58.87 MPa to 50.06 MPa with an increase in the amount of cenospheres. The result was consistent with the conclusion reported in literature [35]. This decrease could be due to an increase in the internal porosity of the LTCC accompanied by an increase in the amount of cenospheres. In addition, the bond strength between the cement paste and cenospheres was lower than the strength of the cement paste, so the increase in the amount of cenospheres could reduce the compressive strength of the LTCC.

Although the compressive strength of the LTCC and the cenosphere content are nonlinear, Figure 7b shows a linear relationship between compressive strength and density. Due to the incorporation of hollow cenospheres, the LTCC was lighter and its void ratio was higher, resulting in lower compressive strength. Table 3 shows that the LTCC had a density of 1.477 g/cm^3^ when it was doped with 30% cenospheres, which was much lighter than the reference group (1.870 g/cm^3^). Therefore, the incorporation of cenospheres significantly reduced the density of the cement-based composites. This could achieve the purpose of reducing the weight of materials.

**Figure 8:**
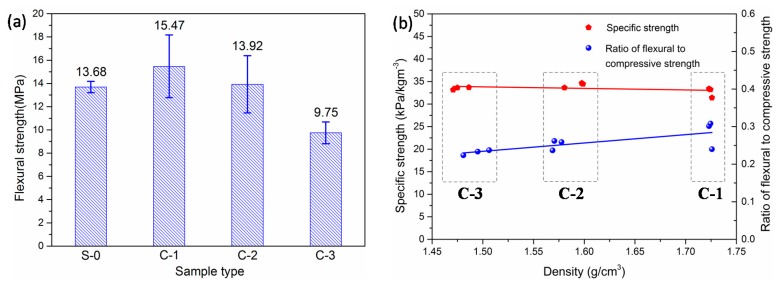
(**a**) Flexural strengths of LTCC. (**b**) Specific strength and ratio of flexural to compressive strength of LTCC with respect to density.

Figure 8a shows the measured 28-day flexural strengths of the LTCCs. It can be seen that the flexural strength of LTCCs incorporating 10% cenospheres was higher than that of the reference group. This phenomenon may be due to the small amount of cenospheres incorporated in the LTCC, which completed the pozzolanic reaction and enhanced the bond strength between the fibers and the cement paste. However, when the amount of cenospheres was more than 10%, the flexural strength of the LTCC continued to decrease as the amount of cenospheres increased. This may be due to the fact that the addition of cenospheres formed an interfacial transition zone between the cenospheres and the cement matrix. Its strength was much lower than the flexural strength of the cement matrix, which resulted in a significant decrease in flexural strength.

Figure 8b shows the specific strength and ratio of flexural-to-compressive strength of the LTCCs with respect to density. Specific strength is the strength of the material divided by its apparent density [36]. It can be seen that the specific strength of the LTCCs was also slightly improved with an increase in the amount of cenospheres. This was because the cenospheres had a low density and strong pozzolanic activity. The ratio of flexural to compressive strength can reflect the toughness of the materials [37]. It can be seen that the ratio of flexural to compressive strength decreased with an increase in the doping amount of cenospheres, which indicated that the incorporation of a large number of cenospheres was not helpful in improving toughness.

#### 3.1.2. Toughness Performance

**Figure 9:**
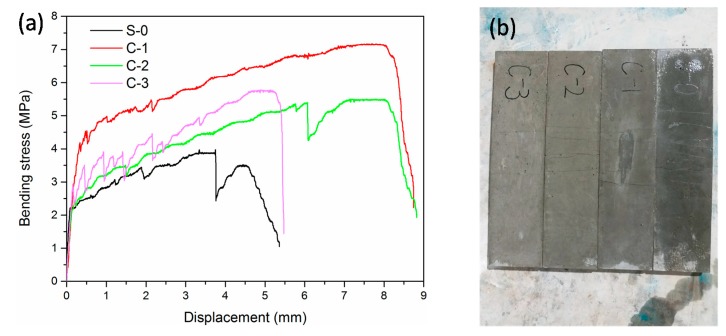
(**a**) Thin plate four-point bending test stress-displacement curves of LTCCs. (**b**) Specimens of LTCCs after the four-point bending test.

The four-point bending test curves of the LTCCs after 28-days of curing are shown in Figure 9a. It was found that the LTCCs incurred obvious strain hardening in the case of lower PVA fiber content, which resulted in lower bending strength. The ultimate bending strength of LTCCs doped with cenospheres was significantly higher than that of LTCCs without cenospheres. The ultimate bending strength and ultimate bending deflection are listed in Table 4. In the four groups of experiments, the ultimate bending strength of the LTCCs incorporating 10% cenospheres was the highest, reaching 6.60 MPa, which was 71.43% higher than that of the reference group. It can be seen that the ultimate bending deformation of LTCCs doped with cenospheres was also higher than that of LTCCs without cenospheres. This indicated that the incorporation of cenospheres was useful in toughening the LTCCs. However, when cenosphere content reached 30%, the LTCC toughness was much lower than that of LTCCs doped with 10% and 20% cenospheres. The reason for this could be that the excessive amount of cenospheres could not undergo s hydration reaction completely, resulting in a decrease in the matrix strength of the LTCCs and the bond strength between the fibers and the cement matrix, which also explained that the multiple falling forces appearing in the stress-displacement curve of C-3 were different from the other curves. The sudden decrease in these forces was the result of the slippage of the fibers in the cement slurry. It can be seen from Figure 9b that the bending specimens of LTCC had obvious characteristics of multi-crack cracking.

#### 3.1.3. Tensile Performance

**Figure 10:**
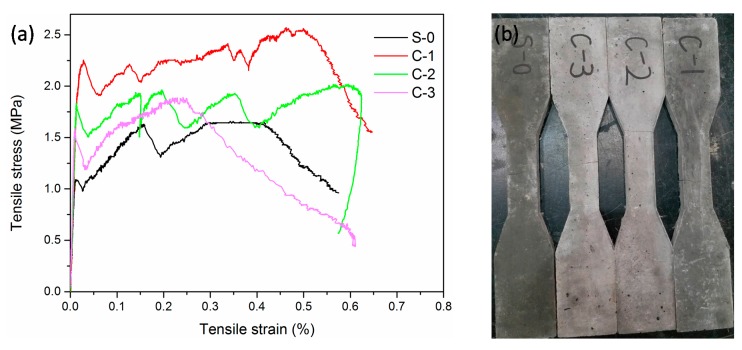
(**a**) Uniaxial tensile stress-strain curves of LTCCs. (**b**) Samples of LTCCs after tensile test.

The uniaxial tensile test stress-strain curves of the LTCCs after 28-days of curing are shown in Figure 10a. The initial cracking stress, ultimate tensile stress, and ultimate tensile strain are listed in Table 4. Similar to the bending test results, the ultimate tensile strength of the LTCCs incorporating cenospheres was significantly higher than that of the reference group. This increase could be explained because the cenospheres have a pozzolanic activity-caused secondary hydration reaction with calcium hydroxide produced by cement hydration to form hydration products, so cenospheres were beneficial to the later strength growth. At the same time, the ultimate tensile strain of the LTCCs doped with 20% cenospheres was 1.525 times that of the reference group, indicating that the incorporation of an appropriate amount of cenospheres was beneficial in improving the tensile properties of the LTCC. As the amount of cenospheres increased, the adhesion between the fibers and the cement slurry decreased, so that more PVA fibers were in the pull-out state rather than being pulled off. This caused a decrease in tensile strength, which also explained the constant decrease in ultimate tensile strength as the amount of cenospheres increased, as shown in the data in Table 4. However, when the amount of cenospheres reached 30%, the tensile strain capacity was minimized. This phenomenon illustrated that when the amount of cenospheres reached a certain level, the adhesion between the fibers and the cement paste was insufficient, resulting in more fibers slipping and a poor tensile strain hardening ability. The tensile specimens only had a few cracks due to the small number of fibers, as shown in Figure 10b. From the perspective of the tensile property, this paper considered that the optimum amount of cenospheres in the LTCC was 20% of the cementitious binders.

### 3.2. Chemical Components

**Figure 11:**
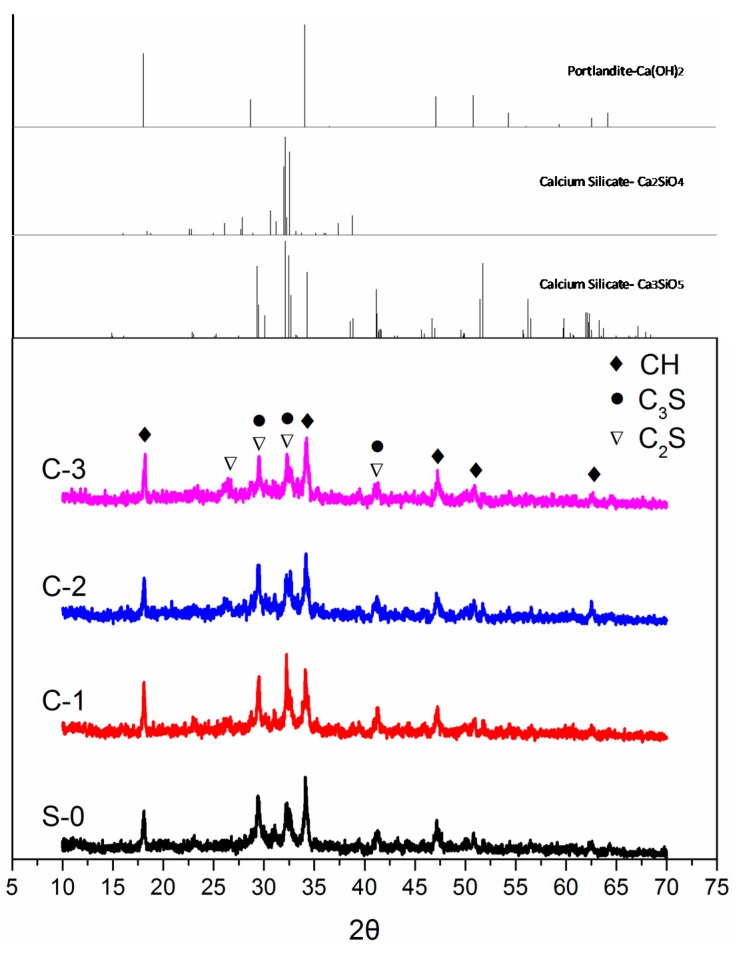
XRD patterns of hydrated LTCCs with different cenosphere content.

To study the effect of cenosphere content on the phase structures of the LTCCs, XRD analysis was performed. XRD results are shown in Figure 11. It was found that C_3_S, C_2_S, and Ca(OH)_2_ were the major components of the LTCCs. Cenosphere content did not affect the LTCC hydration product type, and only the diffraction peak intensities of the products were different. During the hydration process, the peak intensity of Ca(OH)_2_ (2θ = 34.2°) in the reference group without cenospheres was the highest. However, Ca(OH)_2_ was adsorbed on the surface of the fibers and the pores between the fibers, causing stress concentration points. The fibers became brittle, which was disadvantageous for the improvement of toughness. This reason also explained the results of the four-point bending test. A diffuse diffraction peak appeared at 25° to 33° in the XRD pattern, which could be inferred to be a C-S-H gel generated by the LTCC hydration reaction. Compared with the reference group without cenospheres, the diffraction peaks of LTCCs doped with 10% cenospheres contained a larger area, which indicated that it produced more C-S-H gels and cenospheres contributed to the LTCC hydration reaction.

### 3.3. Microstructures

**Figure 12:**
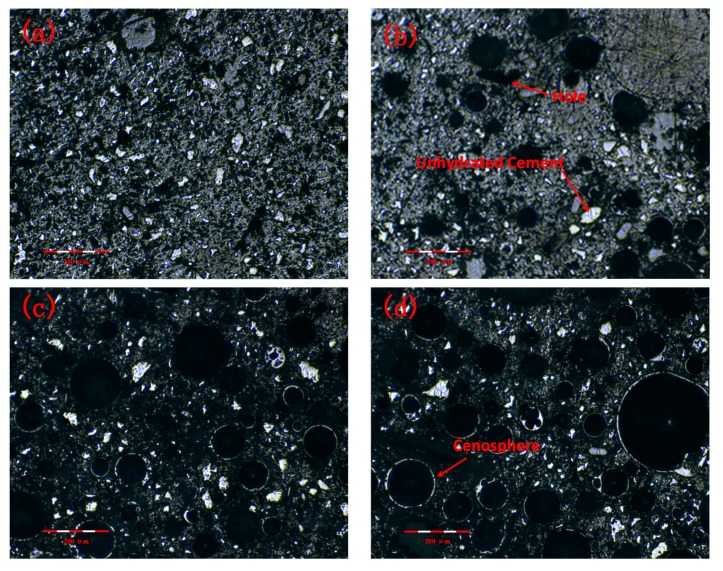
Optical microscope image of LTCCs: (**a**) S-0, (**b**) C-1, (**c**) C-2, and (**d**) C-3.

To observe the microstructural characteristics and hydration products of the LTCCs, an optical microscope and SEM were used at the same scale. Figure 12 shows the distribution of the cenospheres in the four specimens. It can be seen that the unhydrated cement particles in the LTCCs were significantly reduced compared with the reference group, which indicated that the cenospheres had higher pozzolanic activity and promoted the hydration reaction of the cement. Furthermore, it can be seen from Figure 12d that the distribution of the cenospheres in the LTCCs was uniform.

**Figure 13:**
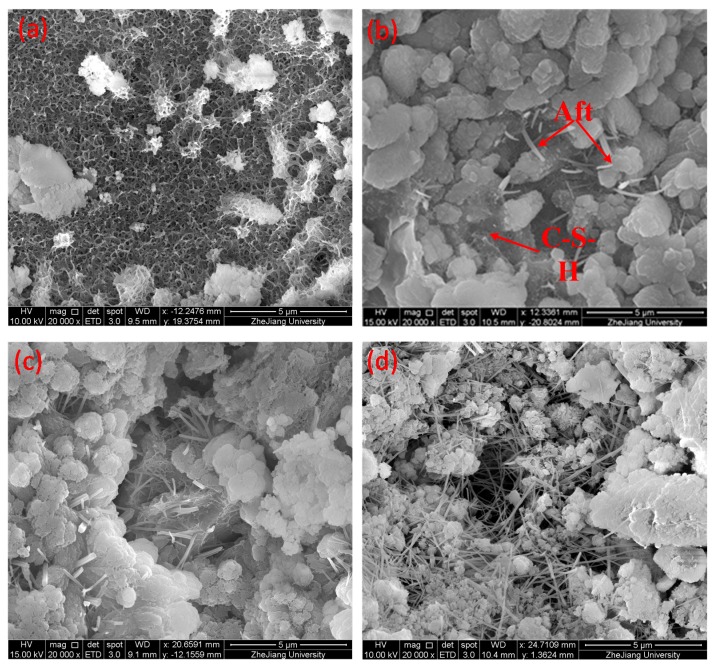
SEM image of hydration product topography: (**a**) S-0, (**b**) C-1, (**c**) C-2, and (**d**) C-3.

Figure 13 shows the microscopic morphology of the hydration products of the LTCCs doped with different amounts of cenospheres. It can be seen that the microstructure of the reference group S-0 was relatively dense and it had much C-S-H gel. However, as the amount of cenospheres continued to increase, the microstructure of the LTCC samples became looser and more and more ettringite (Aft) appeared. The ettringite is a calcium aluminum sulfate formed by the reaction of tricalcium aluminate and gypsum in cement, which had a needle-like appearance [38]. This may be due to the fact that the high content of cenospheres led to a decrease in the content of cement, so the hydration reaction produced less C-S-H gel and could not fill the pores between the ettringites [39,40]. Therefore, the ettringite was exposed and the microstructure of LTCC was relatively loose.

**Figure 14:**
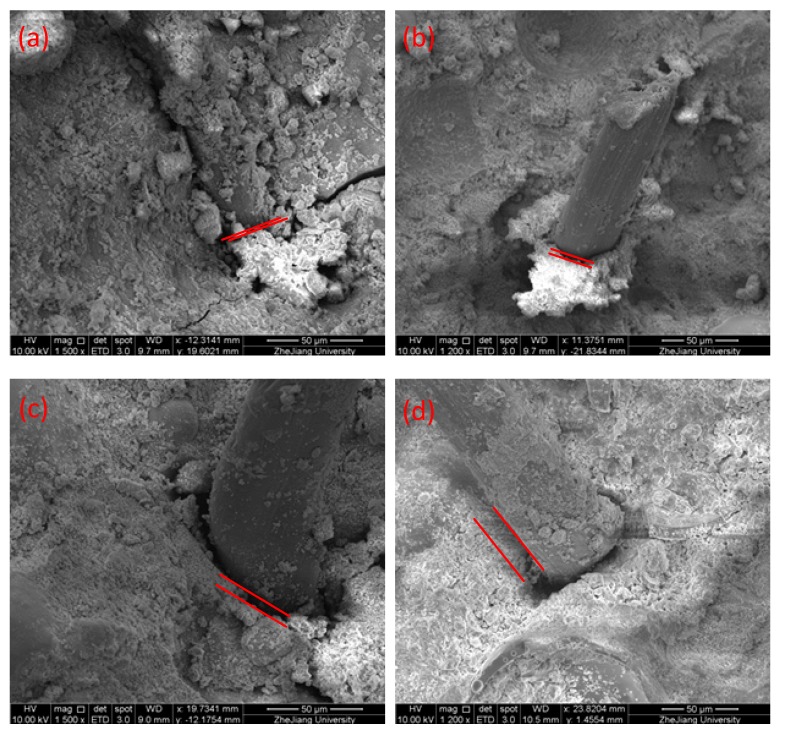
The bonding morphology of fiber to matrix: (**a**) S-0, (**b**) C-1, (**c**) C-2, and (**d**) C-3.

The interfacial adhesion between fiber and hardened cement paste was one of the important factors affecting the macroscopic mechanical properties of the materials [41]. To observe the adhesion of the fiber to the cement paste, SEM was performed on the fracture of the bending specimens. Figure 14 shows that the bonding strength between fiber and cement paste decreased with an increase in the amount of cenospheres. The reason for this phenomenon could be that the incorporation of cenospheres made the microstructure of the cement slurry loose, resulting in large pores at the interface between the fiber and the cement paste. Figure 13 also confirmed this point. The cement paste with weaker bonding strength would cause the fiber to slip, and proper slip is beneficial in large bending deformation. In addition, it can be seen that the fiber surface of the LTCCs was attached with less hydration product than the reference group, indicating that its bonding strength was weaker.

After the cement paste was loaded in the initial crack, the bridging of the fibers acted [42], so the load-displacement curve of the bending test could exhibit a certain strain hardening phenomenon. However, the bonding strength between the fibers and the cement paste in the C-3 sample was too low, which caused the fibers to be pulled out, which decreased its bending strength and bending strain capabilities.

### 3.4. Micromechanics

**Figure 15:**
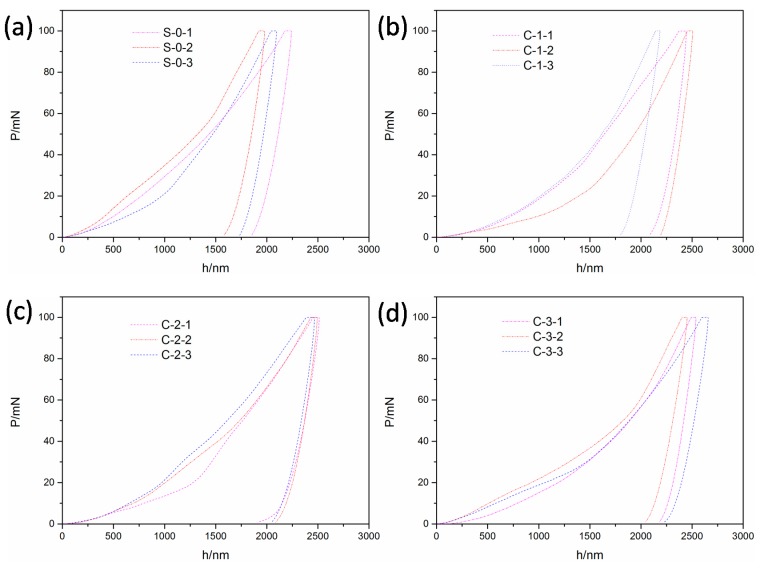
Indentation load-depth (P-h) curves of the matrix phase: (**a**) S-0, (**b**) C-1, (**c**) C-2, and (**d**) C-3.

Figure 15 shows the load-depth curves of the cement matrix of the LTCCs and the reference group. There were some deviations in the initial phase of loading. This was probably because the cement matrix contained hydrated particles, hydration product, and pores, etc. [43], and the indenter easily hit the pores in the process of pressing. However, the unloading curves of the measuring points on the cement matrix were basically parallel and consistent, which verified the reliability of the data. It can be seen from the figure that the depth of the indentation increased with an increase in the cenosphere content, because the microstructure of the cement matrix became looser as the amount of cenospheres increased, as shown in Figure 13. It was easier to press the indenter into the cement matrix.

The calculation results of the elastic parameters of the cement matrix are shown in Table 5. The elastic modulus of the cement matrix was between 28.61 and 38.90 GPa and the hardness was between 0.682 and 0.940 GPa. This was consistent with the results reached by Mondal et al. [44], which concluded that the elastic modulus of the cement hydration product was between 22.89 and 41.45 GPa. The elastic modulus of unhydrated particles was considered to be greater than this interval, and the elastic modulus of hole defects were considered to be less than this interval.

**Figure 16:**
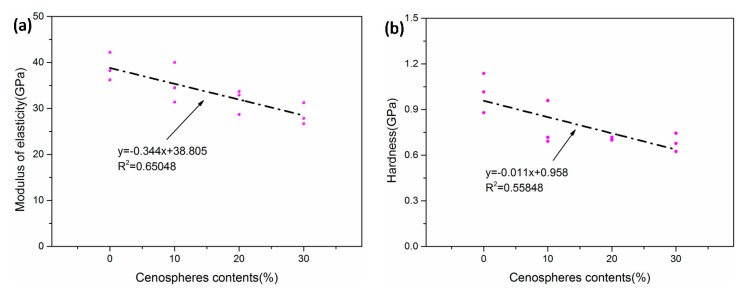
(**a**) Modulus of elasticity with respect to cenosphere content. (**b**) Hardness with respect to cenosphere content.

Figure 16 shows that the modulus of elasticity and hardness of the cement matrix had a good linear relationship with the amount of cenospheres. This was consistent with the law that the compressive strength decreased as the amount of cenospheres increased, which indicated that the hardness of the cement matrix was closely related to the compressive strength.

**Figure 17:**
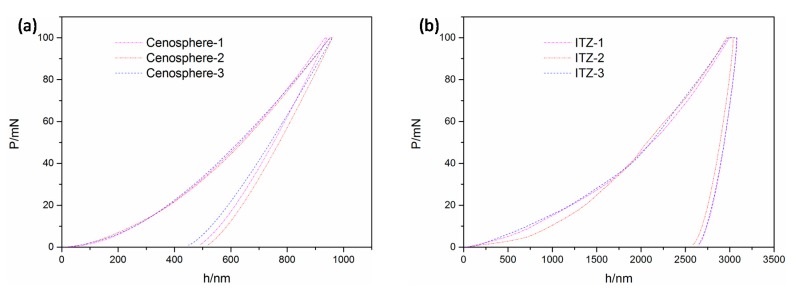
Indentation load-depth (P-h) curves of different phases: (**a**) cenospheres, (**b**) ITZ.

**Figure 18:**
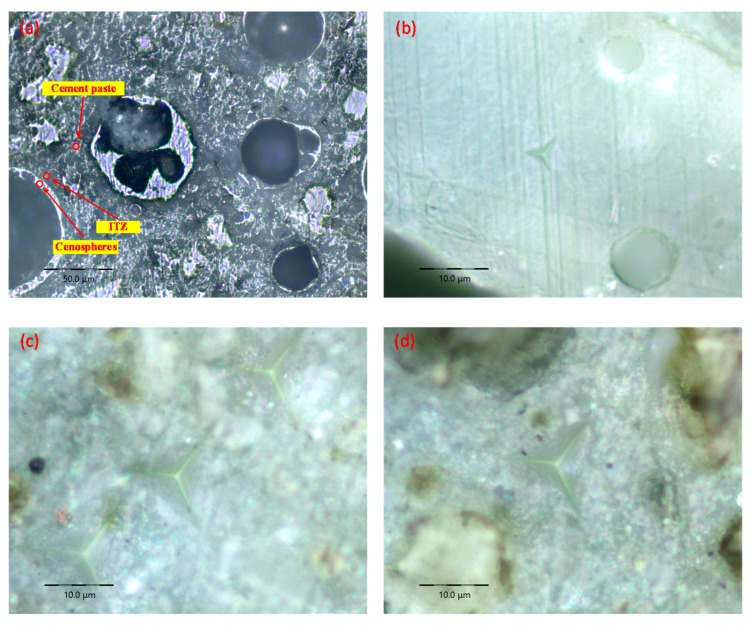
Optical microscope images: (**a**) LTCC, (**b**) cenospheres, (**c**) ITZ, and (**d**) cement matrix.

The interfacial transition zone (ITZ) between the cement paste and the aggregate was loose and porous due to the wall effect, which was considered to be the weakest part of the concrete. Its width was generally 15 to 20 μm [45]. To explore whether the decrease in compressive strength of the LTCCs was related to the interface transition zone, the C-3 sample was selected for nano-indentation testing of the cement matrix, cenospheres, and interface transition zone, respectively; the nano-indentation positions of this testing in the LTCCs are shown in Figure 17. Figure 18a shows the load-depth curves of the cenospheres and the interface transition zone. It can be seen that the maximum indentation depth of the interface transition zone was much deeper than that of the cenospheres, which could also be observed in the indentation morphology of the three different phases shown in Figure 18b–d. The elastic modulus and hardness of the cenospheres were calculated to be 61.81 GPa and 8.09 GPa, respectively, as listed in Table 5. The elastic modulus and hardness of the interface transition zone were only 22.48 GPa and 0.465 GPa, both of which were smaller than that of the cement matrix. These results indicated that the cenospheres are the reinforcing phase in the LTCCs, and the reason for the decrease in compressive strength of the LTCCs was the loose interfacial transition zone between the cenospheres and the cement matrix. Therefore, improving the performance of the interface between the cenospheres and the cement matrix should be further studied in the future.

## 4. Conclusions

Mechanical properties of lightweight toughness cement-based composites (LTCCs) affected by the amount of cenospheres was investigated. Based on XRD, SEM, OM, and NI tests, chemical component, microstructures, and micromechanics of the LTCCs were investigated. Based on these experimental results in the present study, the following conclusions can be drawn:

(a) The compressive and flexural strength of LTCCs decrease with increasing cenosphere content, and the compressive strength decreases linearly with the decrease in density. However, the specific strength of LTCCs increases with increasing cenosphere content. LTCCs have proven to be lightweight and high-strength green cement-based composites.
(b) Both the bending and tensile behavior of LTCCs show significant strain hardening according to the four-point bending and uniaxial tensile curves. The LTCCs with 20% cenosphere content (the ratio of cenospheres to cementitious materials) has excellent toughness and ductility.
(c) The incorporation of cenospheres contributes to the hydration reaction of the LTCC and the production of more C-S-H gel by hydration reaction due to its high pozzolanic activity.
(d) The incorporation of cenospheres causes the microstructure of the LTCCs to become looser and more ettringite to appear. The bonding strength between fiber and cement paste decreases with increasing cenosphere content. A suitable amount of cenospheres is beneficial for fiber slipping to produce large bending deformation and good ductility.
(e) The incorporation of cenospheres decreases the elastic modulus and hardness of the LTCC cement paste; these decreases are linear with the amount of cenospheres. Cenospheres act as a reinforcing phase, and the reason for the decrease in the compressive strength of the LTCCs is the weak interfacial transition zone between the cenospheres and the cement matrix.

## Figures and Tables

**Table 1:** Chemical compositions (wt.%) of the raw materials.

Description	SiO_2_	Al_2_O_3_	MgO	SO_3_	K_2_O	CaO	Fe_2_O_3_	CaCO_3_	Na_2_O
Cement	13.23	4.28	1.69	2.24	1.59	63.95	4.02	8.23	-
Cenosphere	60.10	28.40	1.50	0.03	3.50	0.80	4.80	-	0.90
Silica fume	92.26	0.89	0.96	0.33	1.31	0.49	1.97	-	0.42

**Table 2:** Mixture proportions of LTCCs.

Mix ID	Weight Ratio of Matrix						Fiber (%)
	Cementitious Binders	Cenosphere	PSP	SRA	CSA	Water	PVA (by Volume)
S-0	1	0	0.002	0.002	0.05	0.32	1
C-1	1	0.1	0.002	0.002	0.05	0.32	1
C-2	1	0.2	0.002	0.002	0.05	0.32	1
C-3	1	0.3	0.002	0.002	0.05	0.32	1

**Table 3:** Mechanical properties of the LTCCs.

Mix ID	Density (g/cm^3^)	Compressive Strength	Flexural Strength	Specific Strength	Ratio of Flexural to Compressive Strength
		(MPa)	(MPa)	(kPa/kgm^−3^)	
S-0	1.870	58.87	10.80	31.49	0.184
C-1	1.724	56.35	16.00	32.69	0.284
C-2	1.592	53.86	13.61	33.83	0.253
C-3	1.477	50.06	11.59	33.89	0.232

**Table 4:** Toughness and tensile properties of the LTCCs.

Mix ID	Ultimate Bending Strength (MPa)	Ultimate Bending Deflection (mm)	Initial Cracking Stress (MPa)	Ultimate Tensile Stress (MPa)	Ultimate Tensile Strain (%)
S-0	3.85	4.57	1.10	1.64	0.40
C-1	6.60	7.17	2.25	2.56	0.50
C-2	5.15	7.37	1.83	2.00	0.61
C-3	5.50	5.38	1.56	1.88	0.25

**Table 5:** Modulus of elasticity and hardness of the LTCCs.

Mix ID	S-0	C-1	C-2	C-3	Cenospheres	ITZ
Modulus of elasticity (GPa)	38.90	35.31	31.78	28.61	61.81	22.48
Hardness (GPa)	0.940	0.789	0.695	0.682	8.009	0.465
